# The Emerging Roles of Surfactant Protein-A in Asthma

**DOI:** 10.4172/2155-9899.1000553

**Published:** 2018-07-16

**Authors:** Alane Blythe C Dy, Sasipa Tanyaratsrisakul, Dennis R Voelker, Julie G Ledford

**Affiliations:** 1 Department of Medicine, University of Arizona, Tucson, USA; 2 Department of Medicine, National Jewish Health, Denver, USA; 3 Asthma and Airways Disease Research Center, Tucson, USA

**Keywords:** Asthma, Surfactant protein-A, Chronic obstructive pulmonary disease, Inflammation, Eosinophils

## Abstract

Asthma remains one of the most common respiratory diseases in both children and adults affecting up to 10% of the US population. Asthma is characterized by persistent symptoms, airway inflammation, airflow limitation and frequent exacerbations. Eosinophils are a key immune cell present in a large majority of asthmatics and their presence and dysregulation are clinically associated with more severe asthma. Surfactant protein A (SP-A) provides a first-line of defense in pulmonary innate immunity by virtue of its role in pathogen opsonization. SP-A is known to specifically bind to *Mycoplasma pneumoniae* (Mp), a pathogen associated with asthma exacerbations, and functions to attenuate Mp pathogenicity and abrogate lung inflammation. In addition, SP-A has been shown to inhibit Mp-induced eosinophil peroxidase (EPO) release, a toxic product that can compromise the integrity of the delicate airway epithelia. We have determined that genetic variation in SP-A2 at position 223 that results in a glutamine (Q) to a lysine (K) substitution alters the ability of SP-A to inhibit EPO release and may offer a mechanistic explanation as to why some SP-A extracted from subjects with asthma is unable to carry out normal immune regulatory functions.

## Epidemiology of Asthma

Asthma is a complex and heterogeneous disease with several underlying pathophysiologies, which can result in a variety of clinical phenotypes. These are partially a result of the chemistry between inflammatory cells and soluble mediators at the molecular level. Due in part to heterogeneity of the disease, it has been challenging to obtain precise numbers to assess the global burden of asthma worldwide. Improved monitoring and standardized measurements are needed in order to collect better quality estimates. However, according to the 2014 Global Asthma Report, approximately 334 million people have asthma worldwide, affecting both adults and children [1]. This chronic disease poses a significant health and economic burden to patients, as well as, on the health care system, where the greatest burden is among children aged 10 to 14 years and the elderly aged 75 to 79 years. It affects more than 24 million people in the United States, about 1 in every 13 people or 19 million adults and 7 million children [2].

In addition, short-term, immediate consequences of asthma exacerbations include missed work or school days and emergency room visits and/or hospital admissions. Also, infection with *Mycoplasma pneumoniae*, an atypical bacterium, is linked to severe, persistent asthma and asthma and chronic obstructive pulmonary disease (COPD) exacerbations. Progressive decline of lung function and airway remodeling are two of the more serious effects of untreated asthma that can result in irreversible asthma. Although prevalence and burden of disease highlight the need for the development of more effective management plans and therapies, the complexity of asthma likewise emphasizes the importance of understanding the cellular and ** molecular inflammatory mechanisms to improve patient response to treatment.

## Asthma and Airway Inflammation

Because the lungs are constantly exposed to air, inhalation of environmental contaminants is inescapable. The evolution of the immune system has enabled the efficient and potent defense against invading pathogens and other external insults. However, with the same efficiency and potency, it can also be harmful to the host while responding against non-pathogenic antigens as in allergy. It is widely accepted that asthma is a consequence of airway inflammation prompted by a type 2 adaptive immune response to inhaled allergens [3]. Allergens or other environmental insults stimulate epithelial cells located at mucosal surfaces to produce cytokines, such as IL-33, IL-25 and TSLP, initiating a type 2 response while inhibiting type 1 responses [4]. Release of these cytokines activates group 2 innate lymphoid cells (ILC2s) and dendritic cells. Whereas ILC2s directly secrete canonical type 2 cytokines IL-5 and IL-13, dendritic cells act as a bridge to the adaptive arm of the immune system by stimulating type 2 T helper cells (T_H_2). T_H_2 cells then produce IL-4, IL-5 and IL-13 to recruit downstream effector cells into the lung tissue. Aside from B cells, eosinophil, neutrophil, basophil and mast cell infiltration in various combinations is a typical resulting pro-inflammatory response. This is often accompanied by bronchial inflammation, airway hyperresponsiveness and obstruction.

Disease severity can range from mild, experiencing symptoms occasionally, to severe, having prolonged or persistent symptoms. Triggers of allergic asthma episodes include allergens like mold, dust and pollen and environmental and chemical pollutants such as tobacco smoke and exhaust fumes. Risk factors include family history (genetics), chronic exposure to irritants and obesity. Although there is no known cure for asthma, it is possible to maintain good control of the disease. This is done through timely clinical diagnosis, a clear understanding of triggers and administration of appropriate preventive or maintenance medication.

## Eosinophilia in Asthma

Originating from multipotent hematopoietic stem cells that commit to the myeloid lineage in the bone marrow, eosinophils develop and mature through the influence of specific transcription factors and cytokines. Cytokines IL-3, IL-5 and GM-CSF provide signals to transcription factors PU.1, GATA-1 and C/EBP, which induce progenitor cells to commit to the myeloid lineage and differentiate into eosinophils. Effects of IL-5 are the most potent and specific to eosinophils, promoting their exit from the bone marrow to enter the peripheral blood and survival in tissues. This subtype of the white blood cell contains pre-formed granules composed of proteins, namely, major basic protein (MBP), eosinophil cationic protein (ECP), eosinophil peroxidase (EPO) and eosinophil-derived neurotoxin (EDN) [5]. These granules are cytotoxic, and when released, can damage tissue and cause dysfunction.

Eosinophils have also been shown to produce extracellular traps containing MBP and ECP in response to bacteria, the complement protein C5a and CCR3 ligands [6]. Here, mitochondrial DNA and granule proteins are released in a reactive oxygen species dependent manner, exerting their anti-microbial activity in the extracellular space. Under homeostatic conditions, the half-life of eosinophils in peripheral blood ranges from 8 to 18 h [7]. It is capable of entering tissues through epithelial cell tight junctions by a process called diapedesis. They primarily reside in tissues, such as the gastrointestinal tract, spleen, thymus, mammary glands and uterus, and can survive for 2 to 5 days [7].

Eosinophilia is characterized by the accumulation of eosinophils in the airway and increased eosinophils in peripheral blood [3]. Eosinophilia is a well-documented phenotype of a large subgroup of asthmatics. Additionally, the number of eosinophils in peripheral blood and bronchoalveolar lavage is associated with disease severity in asthmatics [3,7,8]. During inflammation following an aeroallergen exposure, cytokines secreted from a type 2 immune response, primarily IL-4/IL-13, provide the signal for production of eotaxins by the epithelial cells, resulting in the recruitment of eosinophils in lung tissue [4,5]. IL-5 is also produced during this type of immune response, essential for eosinophil proliferation and activation [5].

Recruitment and trafficking of eosinophils are predominantly mediated by the eotaxins, a group of chemokines that act by binding to the cell surface receptor CCR3 [7]. There are three known eotaxins produced in humans, eotaxin-1, −2 and −3 [5,9]. Eotaxin-1 is produced first and is responsible for the early recruitment of eosinophils, while eotaxin-2 and −3 are associated with eosinophil infiltration at 24 h. Other mediators that have chemotactic activity on eosinophils are arachidonic acid metabolites, MCPs and RANTES [5]. Adhesion molecules also play an important part in eosinophil movement. In the allergic airway, eosinophils upregulate LFA-1 and VLA-4 to bind to endothelial cells via ICAM-1 and VCAM-1, respectively [5,7]. Thus, efficient eosinophil migration upon exposure to external stimuli involves three distinct components: production of cytokines by type 2 cells, secretion of chemokines in response to type 2 cytokines and the upregulation of adhesion molecules causing enhanced binding to the endothelium enabling entrance to the airway [5].

Eosinophil activation has been shown to be a key contributor to mucus production, bronchoconstriction and airway inflammation in allergic asthma [10]. Release of proteins, such as major basic protein (MBP) and eosinophil peroxidase (EPO), through degranulation, has the potential to cause airway mucosal damage and remodeling [11]. MBP, for example, acts on vagal muscarinic M2 receptors to cause smooth muscle reactivity; these granules can also cause mast cell and basophil degranulation [5]. Additionally, eosinophils have been shown to act as antigen presenting cells to T cells by expressing MHC class I and II peptides and costimulatory molecules CD28, CD40, CD8 and CD86 [12]. They secrete cytokines that enable polarization of T cells to either the T_H_1 or T_H_2 pathway [5]. Following allergen exposure, eosinophils can internalize and process antigen from the airway and traffic to the draining lymph node to present and activate CD4^+^ T cells. Hence, eosinophilia has been hypothesized to be a significant upstream mediator of disease pathogenesis and suppression of eosinophilia diminishes the airway inflammation associated with asthma [13].

The clearance of eosinophils can be accomplished by inhibiting survival factors and engaging of pro-apoptotic pathways via cell-death receptors [7]. This is a crucial event that is important for the resolution of airway inflammation caused by eosinophilia [14]. Mitochondrial, caspase 8 or caspase 9 activation activates caspase 3 leading to apoptosis [7]. Cell surface receptors implicated in initiating downstream apoptotic pathways include some members of the TNF receptor family, Fas, and Siglec-8 (human) or Siglec-F (murine). Additionally, Bax, a Bcl-2 family member, and TGF-β are molecules that have the ability promote apoptosis and inhibit survival effects, respectively. These are potential targets that can be exploited in inducing eosinophil apoptosis and cell death, which can aid in the resolution of airway inflammation.

## Pulmonary Surfactant Protein-A (SP-A)

The main constituent of the lipoprotein complex that is pulmonary surfactant is lipids, making up 90% of its mass. This includes dipalmitoyl phosphatidylcholine (DPCC), the most abundant lipid in mammalian surfactant and the primary component responsible for its surface-tension reducing ability, other phosphatidylcholines, phosphatidylglycerols and phosphatidylinositols [15]. The remaining fraction constitutes surfactant proteins A, B, C and D (SP-A, SP-B, SP-C and SP-D). SP-A and SP-D are hydrophilic, while SP-B and SP-C are hydrophobic. Also, SP-A and SP-D are part of immune defense mechanisms in the alveolar space, while SP-B and SP-C contribute to the physical role of surfactant.

**Figure 1:**
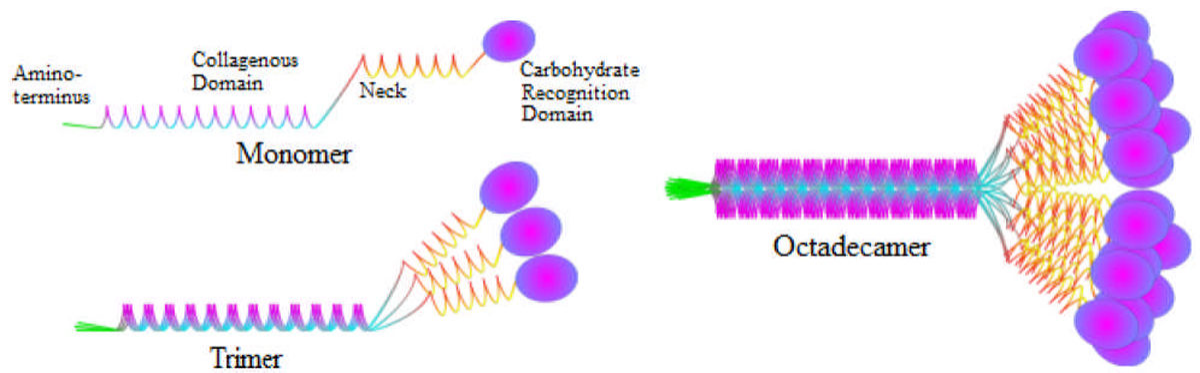
Schematic representation of SP-A. SP-A is comprised of an N-terminal region with a secretion signal peptide (displayed in green), a collagenous domain, a neck region and a carbohydrate recognition domain (CRD). The monomeric form of SP-A initially oligomerizes to become a trimer. Six-trimeric subunits further oligomerize to form a highly complex octadecamer (18-mer) bouquet-like structure. The octadecamer is comprised of gene products from SP-A1 and SP-A2.

One of the hydrophilic proteins, surfactant protein A (SP-A), is the most abundant protein of the four types. It is a lung collectin that has similar structure and function as C1q and mannose-binding lectin (MBL), which are important innate immune defense molecules. Cells in the airway are known to also secrete SP-A [16], independent of phospholipid synthesis [17]. Two genes, SP-A1 and SP-A2, encode human SP-A (hSP-A) and are located on the long arm of chromosome 10. Its translational product is a 248 amino acid long protein that has four distinct domains. As shown in Figure 1, these four domains are 1) N-terminus, 2) collagen tail, 3) neck domain and 4) C-terminal lectin domains (CRD). The N-terminal region contains a 20-amino acid segment that functions as the signal peptide for the translocation of the protein from the rough endoplasmic reticulum to be secreted from the cell and is subsequently lost in this process [18].

As part of the post-translational modifications, SP-A forms disulfide bonds between cysteine residues and, along with other noncovalent interactions, enable oligomer formation [19]. Two of these intermolecular disulfide bonds are located in the CRD, between cysteine residues at position 155 and 246 and at position 224 and 238. In bronchoalveolar lavage fluid (BALF), SP-A forms a 650-kDa octadecamer of six trimers, each trimer believed to be formed by two SP-A1 and one SP-A2 molecules [19]. This octadecamer has a globular head, referred to as the carbohydrate recognition domain (CRD), and a collagenous tail, which forms a structure reminiscent of a bouquet of flowers. This structure is highly conserved across a number of mammalian species: human, dog, rat, mouse, rabbit and pig [18].

## SP-A and Asthma

Collectins, as a family, have the ability to bind and opsonize pathogens for clearance by phagocytes, such as macrophages. Not surprisingly, SP-A has been shown to have this capability through a number of mechanisms [16]. Several molecular structures found on surfaces of fungi, viruses and bacteria can act as ligands for SP-A. Allergens, such as house dust mite and pollen, can also be bound by SP-A to promote their removal [20]. Additionally, SP-A can interact with apoptotic cells for clearance and can regulate production of inflammatory mediators [16]. Enhanced phagocytosis by alveolar macrophages, the resident immune defense cells in the lung and its production of cytokines are heavily influenced by SP-A [20]. Several receptors bound by SP-A have also been identified. The globular heads of the CRD recognize and bind pathogen-associated molecular patterns and the collagen tails interact with receptors on phagocytes for pathogen uptake [21]. This is also the mechanism used for recognition and presentation of apoptotic cells for clearance. There have been reports that SP-A can up-regulate and down-regulate, enhance and inhibit production of specific mediators. This has led to studies that confirmed that SP-A is capable of this dual function of modulating pro- and anti-inflammatory responses depending on its binding orientation and the context of the microenvironment [16,21].

Work by Pastva et al., found that in a mouse model of allergic airways disease, SP-A influences the prevalence, types and functions of CD4^+^ T cells in the lungs and that lack of SP-A enhances the severity of inflammation in conditions like asthma [22]. These studies provide a vital link between SP-A in the adaptive, as well as the innate immune systems as has been previously suggested [23]. Also, SP-A derived from asthmatics was dysfunctional in mediating inflammation compared to SP-A derived from non-asthmatics [24] and it has been suggested that dysfunction may be a consequence of an alteration in the SP-A1:SP-A2 ratio [20]. Since SP-A1 and SP-A2 exert differential effects on alveolar macrophages, a potential mechanism of the persistent inflammation in asthmatics proposed is that the altered SP-A ratio is contributing to the defect in macrophage function [20].

Furthermore, we have recently found that in a cohort of patients with mild to moderate asthma when stratified according to body mass index (BMI) into lean, overweight and obese groups, decreased levels of SP-A were detected among obese asthmatics compared to lean normal and lean asthmatic individuals [25]. Additionally, obese asthmatics have been shown to have higher tissue eosinophilia compared to non-obese asthmatics [26,27]. In support of these findings, using allergic mouse models in mice lacking SP-A (SP-A^−/−^), we discovered SP-A^−/−^ mice had more severe tissue eosinophilia compared to wild-type mice which was resolved upon treatment with exogenous SP-A [25].

## SP-A and *Mycoplasma pneumoniae*

SP-A binds to many pathogens through the carbohydrate recognition domain in a calcium-dependent manner. The binding of SP-A to the pulmonary pathogen, Mp, occurs via two distinct mechanisms: by interaction with the lipid disaturated phosphatidylglycerol [28] and through a specific binding protein, termed MPN372 [29]. While SP-A added to cultures of Mp attenuates growth, this bacteriostatic effect is lost when the lipid dipalmitoylphosphatidylglycerol is added [28]. This finding shows that SP-A has a direct role in immunity to Mp by interacting with lipid ligands expressed on the Mp surface that is independent of an antibody-mediated immune response. Consequences of Mp infection without functional SP-A has also been demonstrated in mouse models in which SP-A null mice have heightened mucus production and inflammation as compared to mice sufficient in SP-A [30,31].

Further studies identified an additional role for SP-A in attenuating Mp-induced eosinophil peroxidase (EPO) release from eosinophils [32]. Interestingly, in addition to the eosinophil or to Mp (as discussed above), we found that SP-A also bound to eosinophils via the FC (CD16/32) receptor complex [32]. Mechanistically, either binding of SP-A to the eosinophil or to Mp was sufficient to limit eosinophil-mediated killing of Mp by preventing EPO release [32].

## Genetic Variation in SP-A Alters Function

The two human SP-A genes have 99% homology [33] and 96% similarity at the protein level [17]. A number of single nucleotide polymorphisms (SNPs) exist, which can translate to missense mutations that may alter their function [17]. Allelic variation of SP-A genes, SP-A1 and SP-A2, have been identified and linked to varying responses to pulmonary infections and control of inflammation [17,34]. The most common amino acid substitutions occur at position 50 and 219 of SP-A1 and position 91 and 223 of SP-A2 [17].

Prior studies have shown that SP-A obtained from asthmatic subjects exhibited diminished ability in controlling *Mycoplasma pneumoniae*-induced mucus and IL-8 production compared to SP-A obtained from normal subjects ex *vivo* [34]. One possibility for this dysfunction could be due to genetic heterogeneity. Indeed, a differential response based on genetic variation with SP-A2 was reported in respiratory syncytial virus infections [35].

**Figure 2:**
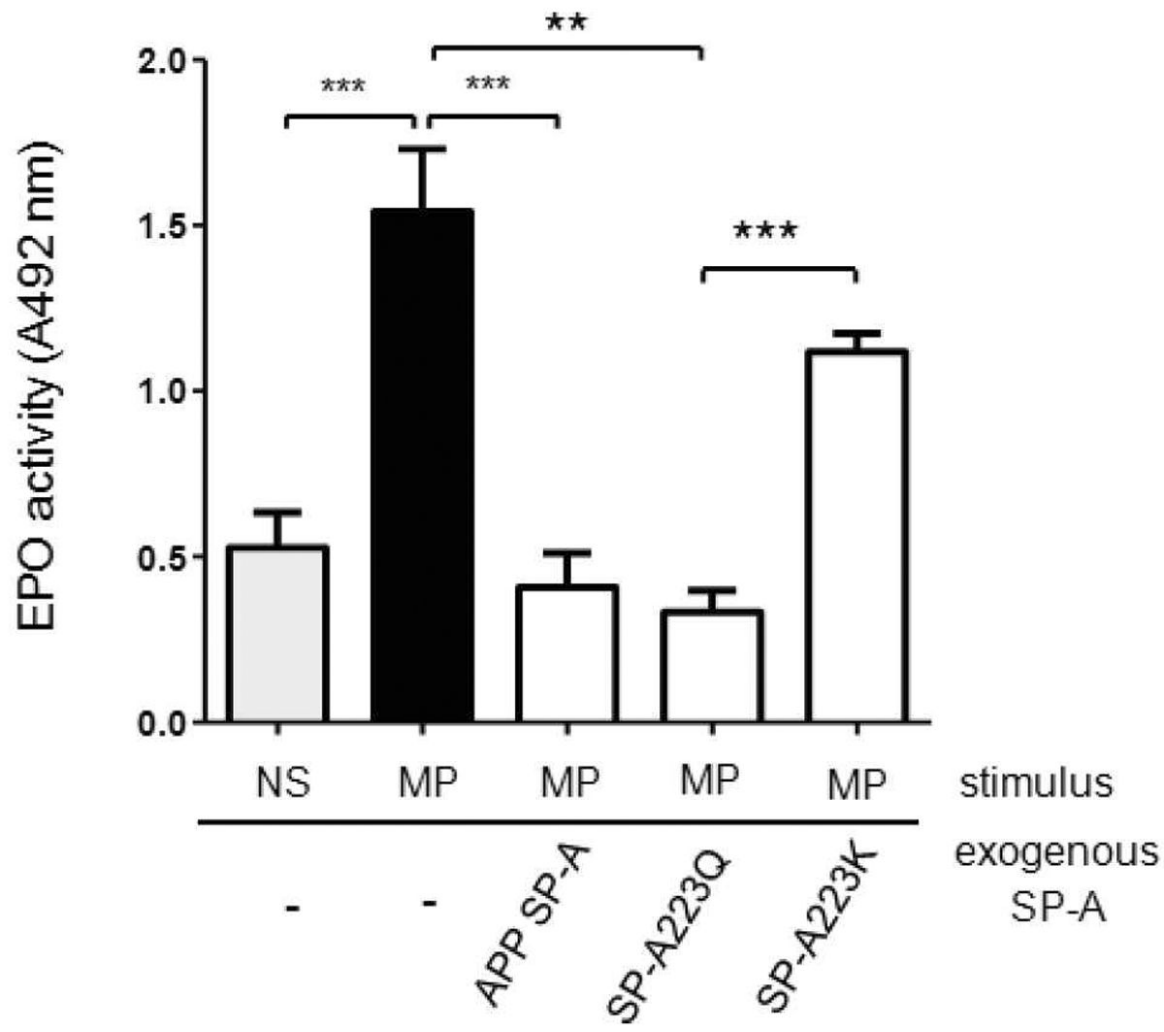
Differential regulation of eosinophil peroxidase release by SP-A2 genetic variants. Purified mouse eosinophils (1 × 10^6^/well) were added to a 96-well plate and incubated for 30 min at 37° C with 5% CO_2_ in the presence or absence of SP-A (25 μg/ml) in PBS. *Mycoplasma pneumoniae* (Mp) was added to the stimulus wells at a concentration of 10 Mp:1 eosinophil. NS is the non-stimulated control. APP SP-A is the positive control that is extracted from BAL of patients with alveolar proteinosis and known to inhibit EPO release from eosinophils as previously described [32]. SP-A223Q and SP-A223K are recombinant human SP-A proteins produced and isolated as previously described [34]. After 1 h of stimulation, supernatants were examined for EPO activity as detected by plate reader at a wavelength of 492 nm. n=mean of 3 separate experiments+SEM. **p<0.01, ***p<0.001.

Interestingly, when we examined the ability of SP-A to inhibit EPO release with rSP-A with either the major (223Q) or minor (223K) allele present in SP-A2, we see a striking and significant difference in activity between the two rSP-As (Figure 2). Such differences in SP-A function dependent on genetic variation in SP-A2 with the presence of either Q or K present at position 223 could offer mechanistic insight to explain why some SP-A is more effective in attenuating phenotypes associated with asthma.

While is it known that cysteine residues within the CRD form intermolecular disulfide bonds between residues at position 155 and 246 and at position 224 and 238, it is not known whether substitution at position 223 from the glutamine (Q) to lysine (K) affects disulfide bond formation or stability of the overall oligomeric protein. Previously, we had determined that 223K rSP-A2 bound to membrane components of Mp better than 223Q rSP-A2 [34]. However, the finding that rSP-A2 223Q is more active in preventing EPO release from eosinophils suggests that the interaction of 223Q rSP-A2 with eosinophils may supersede the binding of rSP-A2 223K for Mp in the mechanistic response and protection from Mp-induced eosinophil degranulation.

## Conclusions

The presence of persistent eosinophilia in the lungs may contribute to symptoms experienced by individuals during an asthma attack or exacerbation. Taken together, several studies suggest that SP-A plays a role in the regulation and control of the host immune response to allergen exposure, as well as downstream inflammatory signal cascades. Moreover, the association between EPO release and genetic variation in SP-A2 suggests an important link between SP-A and the modulation of eosinophils, an immune cell associated with Type 2 asthma and asthma severity.

All data thus far from the field have suggested that an adequate pool of functional SP-A is a necessary contributor for normal lung function, whether during periods of homeostasis, infection or allergen challenge. Decreased levels of SP-A as seen in obese asthmatics [25] or dysfunctional SP-A as detected in some asthmatics [24], have both been associated with altered lung function and may result in enhanced airway inflammation in asthma. We provide evidence that genetic variation within SP-A2 alters the ability of SP-A to inhibit eosinophil EPO release, which could lead to worse asthma exacerbations upon pathogen infection in those asthmatics harbouring the minor allele (223K).

## Methods for EPO Assay

Eosinophils were isolated from the blood of IL-5 transgenic mice (NJ1638; which were a kind gift from the late Dr. James J. Lee, Mayo Scottsdale, AZ) and EPO assay performed as previously described [32]. All mice used in experiments were on protocols approved by the Institutional Animal Care and Use Committee. SP-A was extracted from human BAL of patients with alveolar proteinosis (APP) and used as the oligomeric positive control for activity against EPO release [32]. Recombinant SP-A2 that contained genetic variation at position 223 with either a glutamine (Q) or lysine (K) residue was produced and purified as previously described [34]. Statistical analysis was done with Prism software.
